# Potential sites for landfill development in a developing country: A case study of Ga South Municipality, Ghana

**DOI:** 10.1016/j.heliyon.2019.e02537

**Published:** 2019-10-15

**Authors:** Michael Kaamo Ayaim, Bernard Fei-Baffoe, Alhassan Sulemana, Kodwo Miezah, Festus Adams

**Affiliations:** a Department of Environmental Science, Kwame Nkrumah University of Science and Technology, Kumasi, Ghana; b Centre for Environment and Population Health, Griffith University School of Medicine, Brisbane, Australia

**Keywords:** Environmental science, Analytical hierarchy process, Landfill siting, Geographic information system, Multi-criteria decision analysis, Solid waste disposal

## Abstract

Landfilling, which sits at the bottom of the waste management hierarchy, is the most employed option for managing waste in many emerging economies. In view of the numerous environmental and public health challenges associated with operation of landfills, proper siting would require inputs that overcome the challenges. This study sought to use Geographic Information System application through multi-criteria decision technique to spatially locate suitable sites that fulfill standard landfill guidelines, for waste disposal. Spatial Analyst extension within ArcGIS software was employed for the suitability analysis. Three processes were involved: (1) digitizing to determine boundaries around built up areas, (2) buffering for proximity analysis in order to generate zones around features such as roads, streams, etc. and (3) overlay analysis to determine areas suitable for landfilling. The findings from this study revealed that about 2.62% of the total area was considered as most suitable for landfilling, 2.74% deemed suitable and a large portion (94.64%), considered unsuitable. The study identified 6 most suitable sites that can be used for landfill development in the study area burdened with urbanization. GIS has been used to determine suitable sites for landfill development. Findings from the study serve as guideline for environmentally friendly landfill siting with efficient land-use planning.

## 1 Introduction

Solid waste management systems are expected to ensure effective means of managing waste for promotion of public health and safeguarding the environment. Yesilnacar et al. [1] indicated that, the processes employed in managing waste are key in ensuring that the public is safe and the environment is protected. One main contribution to solving the challenge in managing solid waste has been and continuous to be the use of improved technology [2]. The application of technologies is seen in areas such as route optimization, automated waste collection [3], optical sorting, mechanized biological treatment [4] and suitability analysis for siting facilities [5]. The improved technologies influence human behaviors and manage the processes of waste generation, collection, treatment and disposal procedures. Contrary to the existing and use of several technological approaches to managing waste in advanced countries, crude methods of managing waste still persist in many developing countries.

Mohammedshum et al. [6] acknowledged the huge implications on the environment, societal health and financial consequences, which arise from poor waste management. The disposal of solid waste can create unpleasant smells and pollute groundwater resources with leachates, causing the transmission of diseases and other environmental threats. This calls for siting waste disposal facilities at a considerable distance to waste generation centers to avert potential environmental threats as noted by Anifowose et al. [7] in their study into waste disposal site selection using remote sensing and geographic information system (GIS). Several other studies have considered GIS as an investigative tool in the assessment procedure for selecting suitable sites for landfill development. Wang et al. [8] determined suitable sites for landfill using spatial technologies and analytical hierarchy process (AHP) in Beijing, China. Their findings indicate areas that are best, good and unsuitable for landfill development. Selection of the suitable sites considered the actual conditions of the study area and calculation of criterial weights using the AHP. Eldrandaly et al. [9], break down the meaning of GIS as a computer-based technology and methodology for collecting, processing, managing, analyzing, modelling and presenting spatial data for a wide range of applications. GIS possesses inherent capabilities which allow it to store, manage, edit and analyze huge volumes of data from different sources [10]. There is limited application of this approach in developing country setting. This study therefore employs a multi-criteria decision analysis in a GIS environment to select suitable sites for landfill development in the Ga South Municipal Assembly of Ghana.

**Fig. 1:**
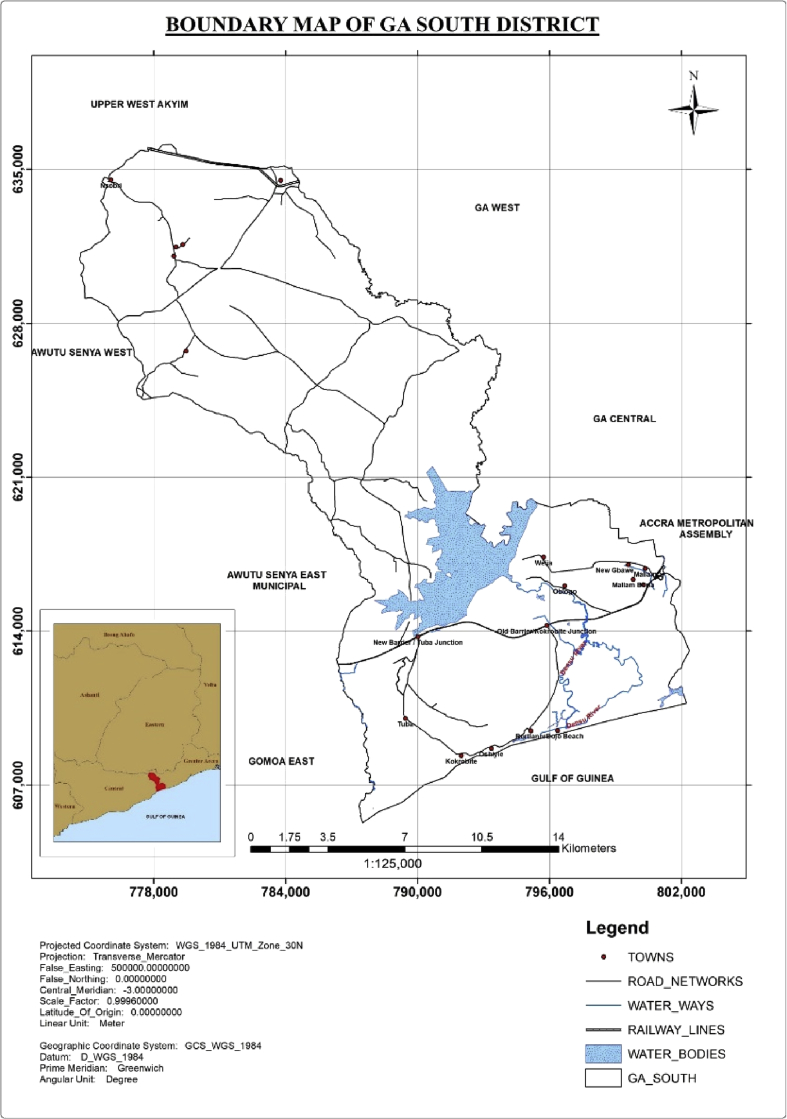
Map of Ga South Municipal Assembly.

Created in late 2007, Ga South Municipal Assembly was one of the four newly formed assemblies which is located in the south western part of Greater Accra Region (Fig. 1). Bounded all about by eight (8) different municipalities, it lies within latitude 5° 33′ 27.9″ N and longitude 0° 18′ 12.3″ W and has an elevation above sea level, estimated to be 72 ft. The municipality covers an approximate area of 342.5 sq. km. It constitutes the Weija-Gbawe, Bortianor-Ngleshie Amanfro and Obom-Domeabra constituencies. The Ga South Municipal Assembly is an industrious city in nature, owing to the increased rate of urbanization over the past few years. This has led to a spike in population numbers which is directly proportional to the volumes of waste generated in the municipality. The cost of managing high volumes of waste with landfilling is normally high. The municipality thus suffers a degree of economic ramifications in the collection, haulage and disposal of waste materials [11].

A dumpsite existed in the Ga South Municipal Assembly (Mallam-Gbawe dumpsite) for waste management purposes but was recently closed down as a result of its inability to meet standard landfill requirements. The need for a dumpsite led to the construction of the dumpsite without effectively considering the economic, health and environmental implications. The disposal site deteriorated and posed dangers to the safety and health of the public, especially the local market. Other consequences associated with poor landfill management is the likelihood of groundwater pollution and environmental degradation as reported by Keestra et al. [12]. Taking note of these factors, Brevik et al. [13] indicated that closure of a landfill site clearly portrays that operation of the facility did not comply with laid down standards. Mekonnen et al. [14] suggest that in order to check these environmental issues, broad consultation is required in waste management. This calls for the application of multi-criteria decision approach when siting landfills.

## 2 Materials and methods

### 2.1 Materials and software

For the purpose of this study, data covering the study area was acquired from different sources. Data on features such as surface water (river/streams), roads and built areas were obtained from Open Street Map^1^. A satellite imagery covering the study area was downloaded from Google Earth. Geological and soil maps were also obtained from the Ghana Geological Survey and the Council for Scientific and Industrial Research respectively. Regarding slope analysis, data was downloaded from the US Geological Survey Global Visualization Viewer website. The software employed were the ArcGIS Version 10.3 and Microsoft Excel 2016 for the preparation, organization and analysis of data.

### 2.2 Methods

Analytical tools in ArcGIS environment such as digitizing, buffering and overlay analysis were applied. Satellite images obtained from Google Earth were digitized to determine the boundaries around built areas. Proximity analysis were conducted to generate zones of given distances using specified criteria from the Ghana Landfill Guidelines [15] as reference (Table 1). Siddiqui et al. [16] suggested that, for final disposal site selection to be in accordance with standard laws and regulations at the local and international levels, a set of generated criteria should be considered for reduced impacts on the economy, society and environment. The study area was characterized by certain features including settlement areas for urban and peri-urban, fault lines, roads which included feeder roads and a highway, different rock formations, a railway line, different elevations and surface water resources. Using the identified features, thematic map layers representing every feature were created for the analysis. For each of the feature under study, buffer zones were created using requirements provided in the Ghana Landfill Guidelines. Table 1 shows the respective buffer distances employed in this study. Proximity analysis was then conducted. This involved the combination of all thematic maps to form a composite map (i.e. the thematic maps were combined in ArcGIS 10.3 to produce a final suitability index map) for further analysis, to determine suitable and unsuitable areas for landfill development.

**Table 1:** Dataset standardization for buffer analysis.

Criteria	Unsuitable (1)	Suitable (2)	Most Suitable (3)
Urban centres	<500 m, >10000 m	3000–10000 m	500–3000 m
Villages and hamlets	<1000 m	1000–2000 m	>2000 m
Waterbodies	<300 m	300–1000 m	>1000 m
Geology	Voltaian and others	Granite	Togo Rocks
Fault	<2000 m	2000–5000 m	>5000 m
Slope	<2%, >15%	10%–15%	2%–10%
Highways	<500 m, > 20000 m	2000–20000 m	500–2000 m
Feeder roads	<100 m	>1000 m	100–1000 m
Railway line	<1000 m	1000–2000 m	>2000 m

The overlay analysis was based on multi-criteria decision techniques using the Analytical Hierarchy Process (AHP) and the Pairwise Comparison Matrix to develop weights. Saaty [17] created the AHP to assist decision makers to make the best choice, in cases where there was the tendency of a conflict between laid down objectives, in this case the criteria. The AHP has been widely accepted and used as a result of its simplicity and flexibility in making decisions [18]. The above-mentioned techniques were used because they are mathematical tools, instrumental in helping decision makers arrive at final solutions that border around complex situations. These methods allow for comparison of different types of factors, taking two items at a time. The pairwise comparison helps compare one criterion to another on a scale of 1–9, resulting in the generation of a matrix for weight determination [19].

Calculating the weights involves three steps as noted by Saaty [17]. In the first step, the criteria were compared, one against the rest at a time. Next step was the summation of the values in each column of the resultant matrix, also known as the normalized comparison matrix. From this, the average value of the elements in each row of the normalized matrix was calculated. These averages depicted an estimated figure of the relative weights of the criteria considered by the study. The Consistency Ratio (CR) was then estimated to determine the level of consistency in the subjective judgements. The weighted sum vector was computed afterwards. In undertaking this, the criteria weights obtained for every column were multiplied by their respective pairwise comparison values (taking a column at a time). In the step that follows, an average value was determined for every row. The values derived were divided by the criterion weights from step 1. λ_max_ is calculated, which represents the mean value of the consistency vector. This value was used to estimate the Consistency Index (CI), to depict the level of departure from consistency (Equation 1).

(1) $$CI=\left(\frac{\lambda -n}{n-1}\right)\;or\;\left(\frac{\lambda -m}{m-1}\right)$$

where;

n = number of alternatives.

m = number of criteria.

The CI was then applied in the calculation of the CR using Eq. (2).

(2) $$CR=\frac{CI}{RI}$$

where RI represents the Random Index, detailed in Table 2.

**Table 2:** Random index table.

n	1	2	3	4	5	6	7	8	9	10
RI	0.00	0.00	0.58	0.90	1.12	1.24	1.32	1.41	1.45	1.49

Saaty [17] indicated that, in the case where the CR < 0.10, then a level of consistency is achieved, whereas if CR ≥ 0.10, there exists a level of inconsistent judgements. Where CR is determined to be 0, then a perfectly consistent pairwise comparison exists. Weighted overlay analysis which involved a construction of a database solely for analyzing data, was carried out, by combining all the input map layers into one, using the weights generated from the AHP and Pairwise Comparison Methods. The model builder in ArcGIS was used in the process to facilitate an easy combination process. The weighted overlay analysis, using the weights generated for each criterion was conducted in ArcGIS environment. The comparisons were based on subjective judgements by the investigators. The weights developed thereof, determined the level of importance of every feature in deciding the final suitability map. A standardization method was adopted to ensure a smooth process in the overlay analysis. According to Drobne [20], this process involves reclassifying values in each criterion to a set membership. A numeric evaluation scale was chosen and for the purpose of this study, a scale of 1–3 was employed, where the values depicted the level of preference, i.e. unsuitable, suitable and most suitable, respectively.

### 2.3 Limitations of study

The study is limited to the Ga South Municipal Assembly, a local authority of Greater Accra Region, Ghana. The methodology is responsive to the environmental criteria used which are intrinsic properties of the study area. This therefore indicates a case study and limits generalization of study findings. Approach can however be followed to conduct environmentally friendly siting of waste management and other hazardous facilities.

## 3 Results and discussion

### 3.1 Thematic maps developed from suitability criteria

**Fig. 2:**
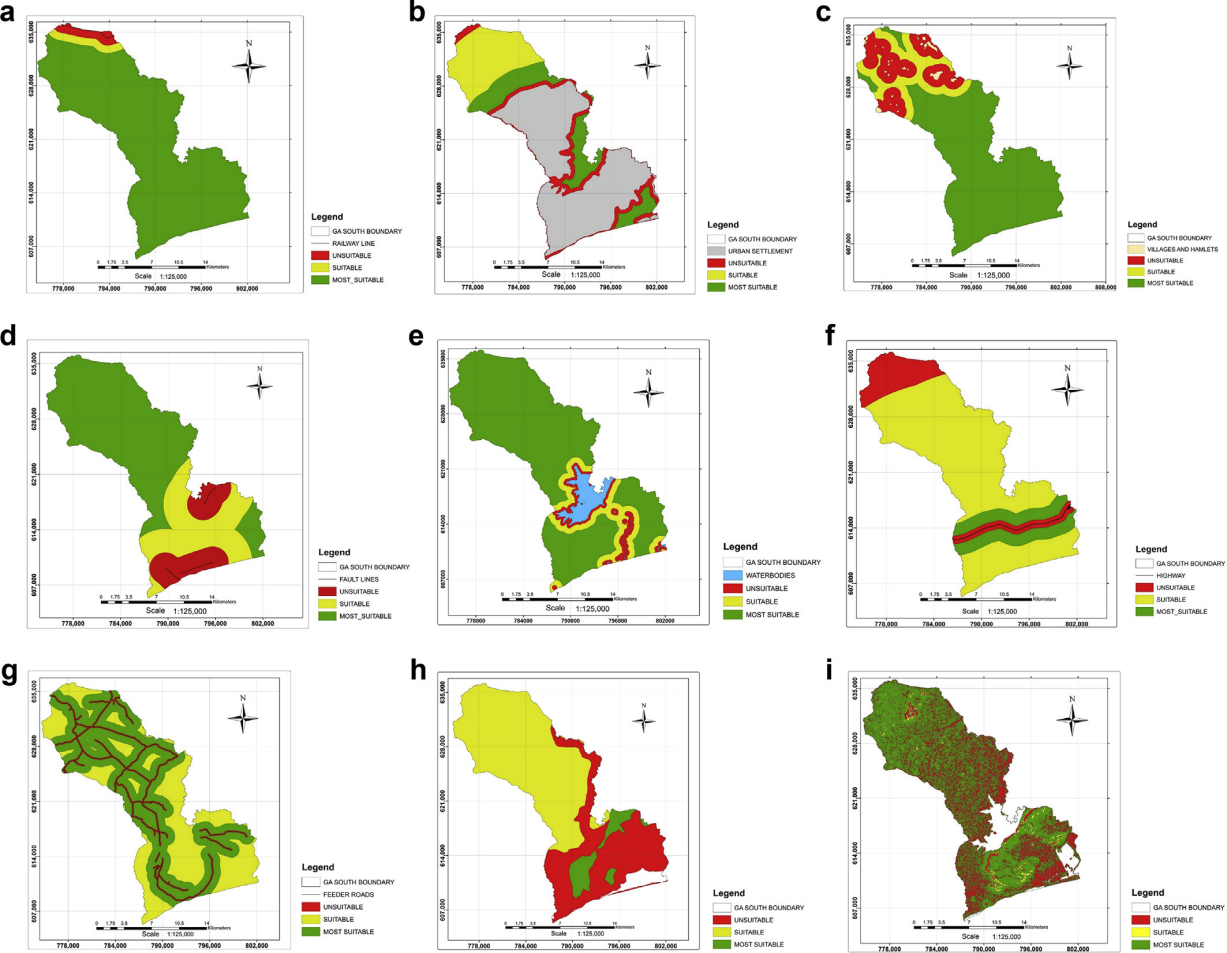
a. Proximity analysis for railway. b. Proximity analysis for urban settlement. c. Proximity analysis for rural areas. d. Proximity analysis for fault line. e. Proximity analysis for waterbodies. f. Proximity analysis for highway. g. Proximity analysis for feeder roads. h. Geological suitability map. i. Slope suitability map.

The thematic maps produced through proximity analysis based on Ghana Landfill Guidelines, using ArcGIS software considered criteria such as railway, built-up area, fault line, waterbodies, road networks, geology and slope as represented by Fig. 2 (a – i).

### 3.2 Weights of suitability criteria

Weights which depicted the level of importance of each criterion under study, were assigned having conducted the AHP and also generated Pairwise Comparison Matrices in the process. This was done to ensure sound decision making. Table 3 depicts the pairwise comparison generated, and the weights that were estimated through the AHP means.

**Table 3:** Pairwise comparison matrix and respective criterion weights.

	CATEGORIES									WEIGHTS
	UC	VH	W	G	F	S	H	FR	R	
Urban centres	1	3	3	4	4	5	6	6	8	**0.293727**
Villages and hamlets	1/3	1	3	3	4	5	4	7	8	**0.213612**
Waterbodies	1/3	1/3	1	3	3	5	5	7	7	**0.168475**
Geology	1/4	1/3	1/3	1	3	4	4	5	5	**0.116897**
Fault	1/4	1/4	1/3	1/3	1	2	3	3	3	**0.069667**
Slope	1/5	1/5	1/5	1/4	1/2	1	2	3	3	**0.051747**
Highways	1/6	1/4	1/5	1/4	1/3	1/2	1	2	2	**0.03753**
Feeder roads	1/6	1/7	1/7	1/5	1/3	1/3	1/2	1	1	**0.025168**
Railway	1/8	1/8	1/7	1/5	1/3	1/3	1/2	1	1	**0.023177**
TOTAL	**2.825**	**5.635**	**8.352**	**12.233**	**16.500**	**23.167**	**26.000**	**35.000**	**38.000**	1

UC = Urban Centers, VH = Villages and hamlets, W = Waterbodies, G = Geology, F = Fault, S = Slope, H = Highways, FR = Feeder roads and R = Railway.

### 3.3 Suitable sites for landfill development

**Fig. 3:**
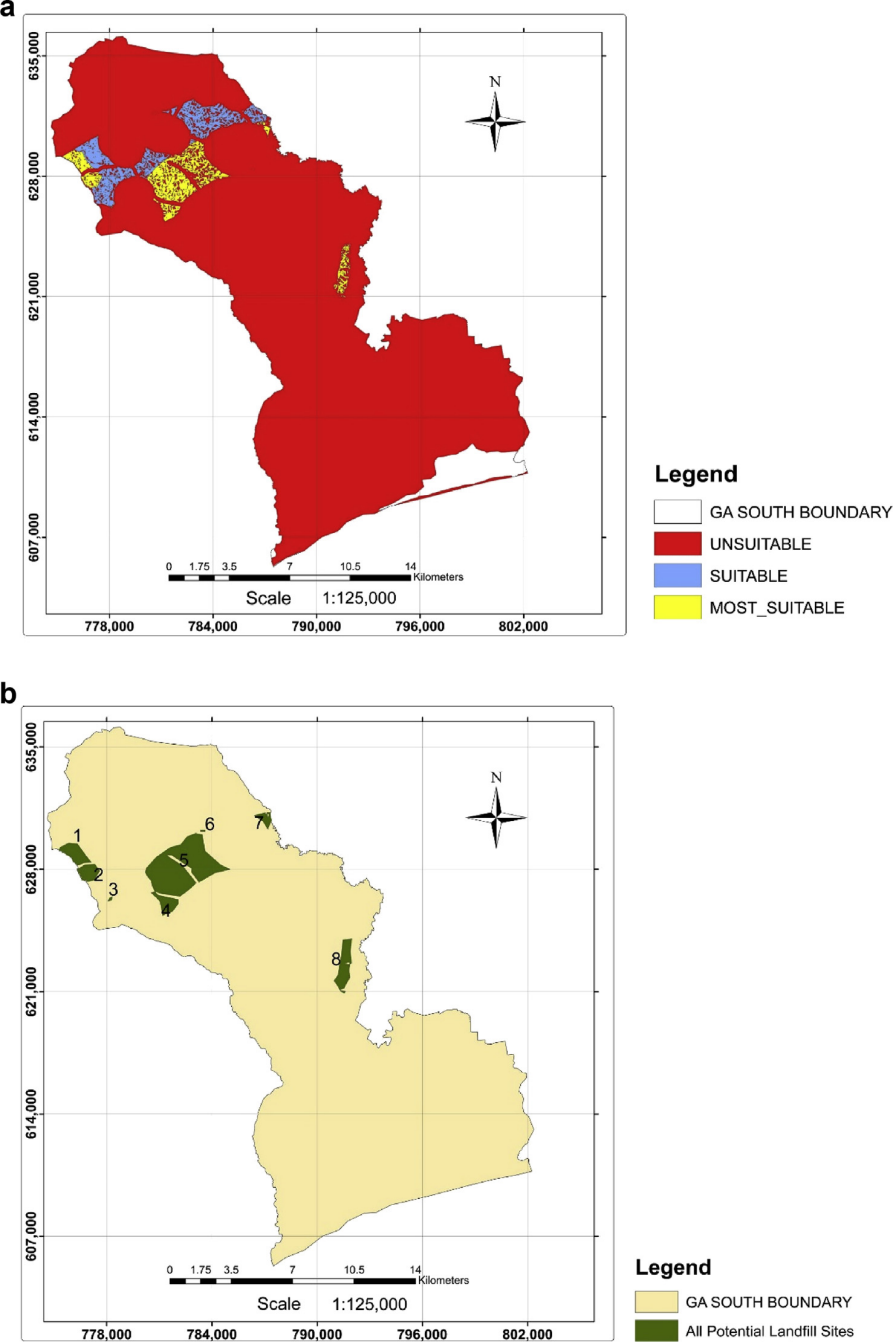
a. Suitability index map. b. Map showing potential sites.

The result obtained in the suitability index map shows 8 prospective sites suitable for landfill development as presented in Fig. 3 (a and b). The findings indicate that a low percentage of the study area is suitable for constructing a landfill facility. In all, 94.64% of the total area, representing 324.06 km^2^ was deemed unsuitable. Suitable sites of 2.74% portion, equivalent to 9.39 km^2^, and 8.95 km^2^ representing 2.62% characterized areas considered as most suitable. Owing to the consequences that are likely to arise in the operation of landfill, the 8 potential sites were subjected to further testing and screening considering a minimum land size requirement of 0.4 km^2^ which fell within 0.1–0.5 km^2^ used by previous studies [21, 22, 23, 24, 25]. The land size can be attributed to the life span of the waste disposal site and expansion projects that are likely to be conducted to assimilate the disposed waste.

### 3.4 Evaluation of candidate landfill sites

**Fig. 4:**
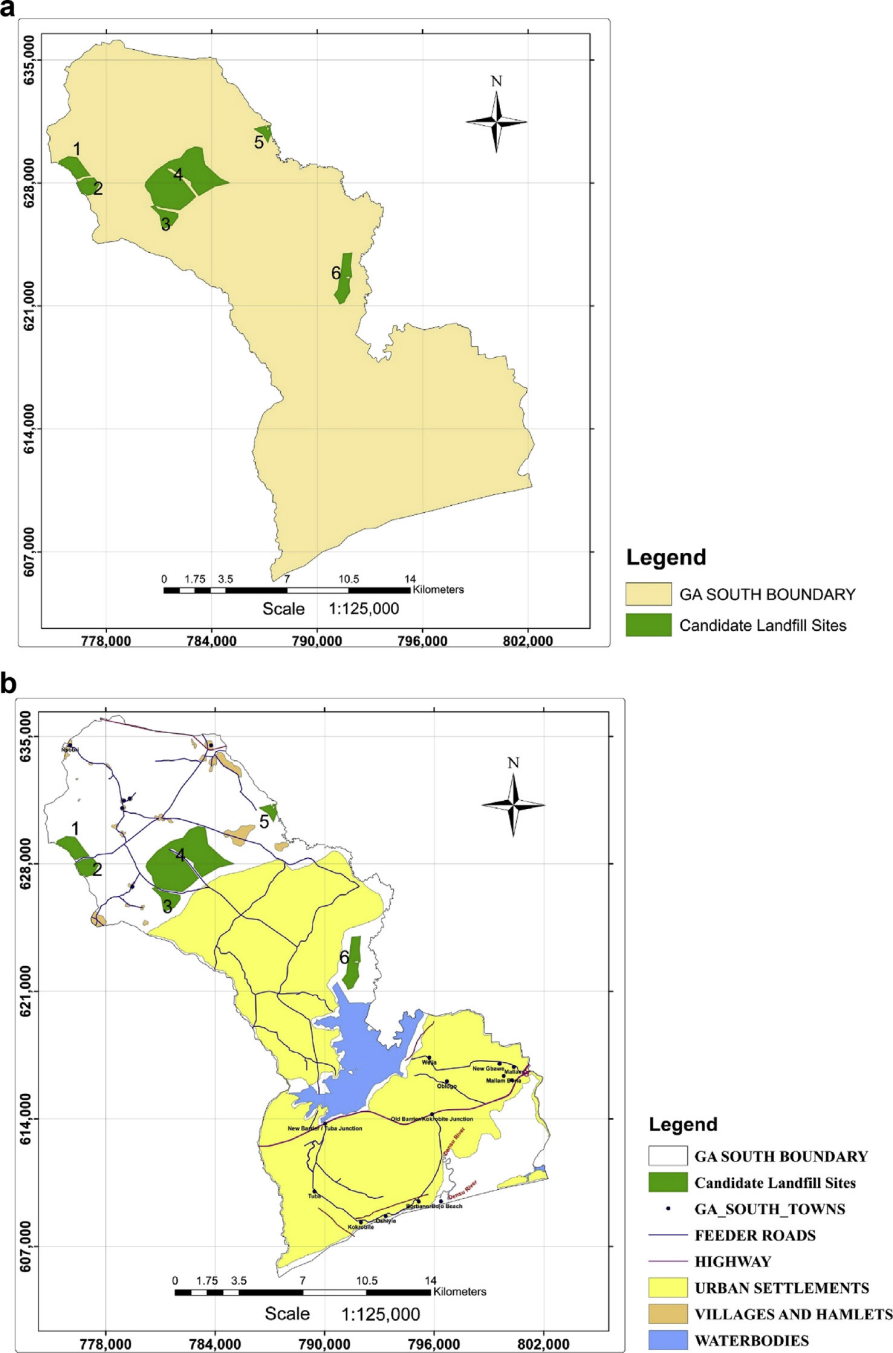
a. Candidate landfill sites. b. Suitable areas for landfills.

After taking out suitable sites which didn't meet the land size requirement using a selection by attribute tool in ArcGIS, 6 sites were eventually selected (Fig. 4a and b). These sites were further ranked using developed criteria by Kabite et al. [22]. These included haulage distance, landfill size and distance to residential areas (Table 4). Having carried out the necessary comparison of the newly developed criteria, the sites were further subjected to AHP calculations and their suitability index scores determined as presented in Tables 5, 6, 7, and 8. From the scores, sites 4, 6 and 3 had positions of 1^st^, 2^nd^ and 3^rd^, respectively, whereas sites 1, 5 and 2 took the 4^th^, 5^th^ and 6^th^, positions respectively. Consideration should be given to these areas by the local authority when siting landfills to ensure that the environment is conserved. The approach can also be used in siting other undertakings with considerable effects on the environment.

**Table 4:** Pairwise comparison on further developed criteria.

	CATEGORIES			WEIGHTS
	LS	DTR	HD	
Landfill size	1	3	3	**0.574**
Distance to residences	1/3	1	3	**0.286**
Haulage distance	1/3	1/3	1	**0.140**
TOTAL:	**1.667**	**4.333**	**7.000**	1

LS = Landfill size, DTR = Distance to residences, HD = Haulage distances.

**Table 5:** Pairwise comparison of alternative landfill sites (landfill size criteria).

	Site 1	Site 2	Site 3	Site 4	Site 5	Site 6	WEIGHTS
Site 1	1	4	4	1/8	6	1/4	**0.141**
Site 2	1/4	1	1/2	1/9	5	1/4	**0.041**
Site 3	1/4	2	1	1/9	5	1/4	**0.060**
Site 4	8	9	9	1	9	8	**0.536**
Site 5	1/6	1/5	1/5	1/9	1	1/4	**0.026**
Site 6	4	4	4	1/8	4	1	**0.196**
TOTAL:	**13.667**	**20.200**	**18.700**	**1.583**	**30.000**	**10.000**	1

**Table 6:** Pairwise comparison of alternative landfill sites (distance to residence criteria).

	Site 1	Site 2	Site 3	Site 4	Site 5	Site 6	WEIGHT
Site 1	1	1/2	1/4	1/4	1/3	1/4	**0.049**
Site 2	2	1	1/3	1/3	1/2	1/3	**0.081**
Site 3	4	3	1	1/2	3	1/2	**0.186**
Site 4	4	3	2	1	3	1/2	**0.257**
Site 5	3	2	1/3	1/3	1	1/3	**0.115**
Site 6	4	3	2	2	3	1	**0.312**
TOTAL:	**18.000**	**12.500**	**5.917**	**4.417**	**10.833**	**2.917**	1

**Table 7:** Pairwise comparison of alternative landfill sites (haulage distance criteria).

	Site 1	Site 2	Site 3	Site 4	Site 5	Site 6	WEIGHTS
Site 1	1	1/2	1/3	1/3	1/2	1/3	**0.060**
Site 2	2	1	1/3	1/3	1/2	1/3	**0.088**
Site 3	3	3	1	1/2	3	1/2	**0.183**
Site 4	3	3	2	1	3	1/2	**0.253**
Site 5	2	2	1/3	1/3	1	1/3	**0.108**
Site 6	3	3	2	2	3	1	**0.308**
TOTAL:	**14.000**	**12.500**	**6.000**	**4.500**	**11.000**	**3.000**	1

**Table 8:** Determination of suitability index scores.

	LANDFILL SIZE (A)	DISTANCE TO RESIDENCES (B)	HAULAGE DISTANCE (C)	A + B + C	RANK
Weighted Scores from AHP	0.574	0.286	0.14	Suitability Index Score	
Site 1	0.574 × 0.141	0.286 × 0.049	0.140 × 0.060	0.103	**4th**
Site 2	0.574 × 0.041	0.286 × 0.081	0.140 × 0.088	0.059	**6th**
Site 3	0.574 × 0.060	0.286 × 0.186	0.140 × 0.183	0.113	**3rd**
Site 4	0.574 × 0.536	0.286 × 0.257	0.140 × 0.253	0.419	**1st**
Site 5	0.574 × 0.026	0.286 × 0.115	0.140 × 0.108	0.063	**5th**
Site 6	0.574 × 0.196	0.286 × 0.312	0.140 × 0.308	0.245	**2nd**

## 4 Conclusion

Selection of a landfill should encompass interdisciplinary collaboration and also the consideration of factors ranging from environmental to social and economic. The findings from this study has revealed that the application of multi-criteria decision analysis tools in a GIS environment is an effective way of arriving at a suitable site for landfill development. As is evident, the AHP procedure adopted in this study provides a flexible approach in dealing with complex issues, which have the tendency to conflict considering there are interactions that exist between set objectives or primary goals. This eventually helps decision makers arrive at solutions in proper siting of landfill. The factors generated to help in assessment of landfill site suitability, should be noted as very fundamental to landfill siting and are globally acknowledged. In spite of this, each research conducted using this approach provides outputs directly relevant to the respective study area in terms of its geographical location and associated properties. The rankings and weights estimated have been determined solely on the basis of conditions that are specific to the study area and the importance each criterion plays on the final outcome respectively. After the suitability analysis, 6 suitable sites which meet both local and international standards were identified and ranked, to help effectively site landfills for waste disposal in the Ga South Municipal Assembly. Multi-criteria decision analysis tools in a GIS environment is therefore recommended as a decision support tool in siting landfills and other environmentally sensitive undertakings.

## Data availability

Data for this study are securely kept by the principal investigator and corresponding author and will be made available upon request.

## Declarations

### Author contribution statement

A. Sulemana: Analyzed and interpreted the data; Wrote the paper.

M. K. Ayaim: Conceived and designed the experiments; Performed the experiments; Analyzed and interpreted the data; Wrote the paper.

B. Fei-Baffoe: Conceived and designed the experiments; Wrote the paper.

K. Meizah: Wrote the paper.

F. Adams: Analyzed and interpreted the data.

### Funding statement

This research did not receive any specific grant from funding agencies in the public, commercial, or not-for-profit sectors.

### Competing interest statement

The authors declare no conflict of interest.

### Additional information

No additional information is available for this paper.

## Footnotes

1 www.openstreetmap.org.
